# Precision Segmentation of Subretinal Fluids in OCT Using Multiscale Attention-Based U-Net Architecture

**DOI:** 10.3390/bioengineering11101032

**Published:** 2024-10-16

**Authors:** Prakash Kumar Karn, Waleed H. Abdulla

**Affiliations:** Department of Electrical, Computer, and Software Engineering, The University of Auckland, Auckland 1010, New Zealand

**Keywords:** Optical Coherence Tomography (OCT), sub-retinal fluids, multiscale attention mechanism, deep learning, U-Net, medical imaging

## Abstract

This paper presents a deep-learning architecture for segmenting retinal fluids in patients with Diabetic Macular Oedema (DME) and Age-related Macular Degeneration (AMD). Accurate segmentation of multiple fluid types is critical for diagnosis and treatment planning, but existing techniques often struggle with precision. We propose an encoder–decoder network inspired by U-Net, processing enhanced OCT images and their edge maps. The encoder incorporates Residual and Inception modules with an autoencoder-based multiscale attention mechanism to extract detailed features. Our method shows superior performance across several datasets. On the RETOUCH dataset, the network achieved F1 Scores of 0.82 for intraretinal fluid (IRF), 0.93 for subretinal fluid (SRF), and 0.94 for pigment epithelial detachment (PED). The model also performed well on the OPTIMA and DUKE datasets, demonstrating high precision, recall, and F1 Scores. This architecture significantly enhances segmentation accuracy and edge precision, offering a valuable tool for diagnosing and managing retinal diseases. Its integration of dual-input processing, multiscale attention, and advanced encoder modules highlights its potential to improve clinical outcomes and advance retinal disease treatment.

## 1. Introduction

Optical Coherence Tomography (OCT) is a medical imaging technique that uses light waves to produce detailed, cross-sectional images of the retina, the clear tissue at the back of the eye. These images assist healthcare providers in diagnosing and tracking various eye conditions, including age-related macular degeneration, glaucoma, and diabetic retinopathy. Optical Coherence Tomography (OCT) images are produced by directing light into the eye and capturing the reflections that return. The strength and timing of these reflections are then utilized to create a detailed visualization of the eye’s internal tissue layers. OCT is a non-invasive, painless procedure that takes only a few minutes to complete. It is frequently combined with other eye examinations, such as fundus photography or visual field tests, to offer a comprehensive assessment of an individual’s eye health.

Retinal diseases, such as Diabetic Macular Oedema (DME) and Age-related Macular Degeneration (AMD), often accumulate fluid in various retinal layers, necessitating precise segmentation for accurate diagnosis and treatment planning. Optical Coherence Tomography (OCT) has become a critical imaging modality for visualizing retinal structures and identifying pathological fluids. Despite advancements in automated segmentation techniques, existing methods often face challenges in accurately segmenting multiple fluid types and maintaining edge precision.

To address these challenges, we aim to develop a robust deep-learning architecture that uses B-scan images from various datasets to segment multiple classes of retinal fluids. Our proposed model introduces a novel approach by generating edge maps of OCT slices and utilizing dual inputs to enhance the segmentation accuracy. This approach is integrated into an autoencoder-based hybrid network, which combines the strengths of Residual and Inception modules within the encoder section. These modules are designed to extract multiscale and multilevel features, significantly improving the performance of semantic segmentation of retinal fluids.

**Figure 1:**
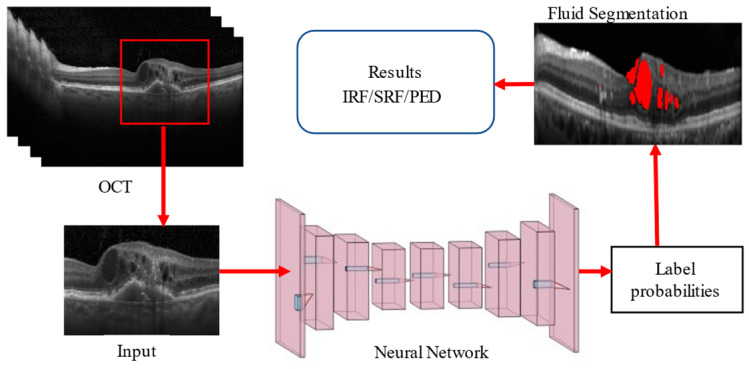
General Procedure of Retinal Fluid Segmentation.

The architectural structure of our proposed network follows the conventional U-Net framework, with key enhancements in the encoder block. The hybrid encoder merges residual and inception networks, processing dual inputs to generate an edge map feature vector. This design ensures the dimensions of the input image and the images after convolution and max-pooling are appropriately matched, reducing the dimensions by two, four, and eight times in successive blocks. The General Procedure of Retinal Fluid Segmentation is given in Figure 1.

In a nutshell, our hybrid deep learning architecture aims to advance the field of retinal fluid segmentation by providing a more accurate and efficient tool, facilitating better clinical outcomes for patients with retinal diseases.

The key contributions of this research article are given below:

**Key Contributions:**

- Innovative Network Architecture: The proposed deep learning model employs a unique encoder–decoder architecture inspired by U-Net, incorporating Residual and Inception modules along with an autoencoder-based multiscale attention mechanism. This design significantly enhances the accuracy and precision of retinal fluid segmentation in OCT images.
- Superior Performance Across Datasets: The model demonstrates exceptional performance on multiple datasets, including the RETOUCH, OPTIMA, and DUKE datasets. It achieves high F1 Scores for various fluid types (IRF, SRF, and PED), indicating robust and reliable segmentation capabilities across different imaging platforms and ground truth annotations.
- Clinical Relevance and Practicality: By improving segmentation accuracy and edge precision, the proposed architecture offers a valuable tool for diagnosing and managing retinal diseases such as Diabetic Macular Oedema (DME) and Age-related Macular Degeneration (AMD). Its integration of dual-input processing and multiscale feature extraction highlights its potential to enhance clinical outcomes and advance retinal disease treatment strategies.

The rest of the paper is structured as follows: Section 2 outlines the literature review. Section 3 describes materials and method used in this research. In Section 4, implementation of the network. In Section 5, performance measures are explained along with results from all datasets presented and analysed. The key conclusions are summarised in Section 6.

## 2. Related Works

Recent advancements in neural network architectures have significantly improved the analysis of Optical Coherence Tomography (OCT) images, particularly in medical diagnostics. A notable contribution is the Generalized Liquid Neural Network (GLNN) framework, which has demonstrated superior performance across various applications [1]. In the context of OCT image analysis, the GLNN framework achieved an impressive F1 score of 0.98, surpassing the classical Liquid Neural Network (LNN) by 10%. This enhancement underscores the framework’s potential for more accurate and reliable diagnostic tools, marking a critical advancement in the application of neural networks to complex medical imaging tasks. Fernández et al. [2] were among the first to present a semi-automated, active contour-based method for segmenting intraretinal fluid (IRF) and subretinal fluid (SRF) from 2D OCT images. However, due to the limitations of semi-automated methods, there has been an increasing need for fully automated approaches. To address this, Wilkins et al. [3] developed an automated technique for classifying cystoid regions of cystoid macular oedema (CME) using a fast bilateral filter for speckle noise reduction and boundary tracing, achieving a sensitivity of 91%. Zhang et al. [4] proposed a graph-cut algorithm guided by an AdaBoost classifier to segment cystoid regions in 3D OCT images, reporting a true positive rate of 84.6%. Similarly, Rashno et al. [5] introduced an innovative method combining neutrosophic transformation with a graph-based shortest path algorithm to segment cystoid regions in diabetic macular oedema (DME).

With the increasing ease of developing new architectures and improved performance, deep learning has become widely adopted for OCT image analysis. For example, an FCNN proposed by [6] classified patch-wise voxels into IRF and SRF. The U-Net architecture, introduced by Ronneberger et al. [7], has become one of the most commonly used models for biomedical image segmentation. Sappa et al. [8] designed RetFluidNet, a modified U-Net incorporating skip connections and Atrous Spatial Pyramid Pooling (ASPP), achieving accuracies of 80.05%, 92.74%, and 95.53% for IRF, pigment epithelial detachment (PED), and SRF, respectively. Additionally, Guo et al. [9] developed RefNet, achieving an F1 score of 0.892 for segmenting retinal fluid using OCT and angiography data.

Retinal conditions encompass a range of serious retinal issues. Choroidal neovascularization (CNV) involves the growth of new blood vessels, which can lead to significant visual impairment. Diabetic macular oedema (DME) is characterized by fluid accumulation that impacts sharp central vision. Drusen are markers that indicate potential age-related macular degeneration (AMD) [10,11]. To analyze OCT images, various advanced machine learning techniques have been employed. These include hybrid attention-based U-Net models and vision transformers, which offer promising avenues for improved diagnosis and treatment [12,13].

Recent literature has introduced innovative methods and datasets for fluid segmentation. For instance, the DUKE DME, OPTIMA, and RETOUCH Challenge datasets have been instrumental in evaluating these techniques. U-Net + + architecture and a Spatially adaptive denormalization Unit with a class-guided module [14] achieved a dice score of 0.78 for segmenting IRF on the DUKE DME dataset. Girish et al. [15] developed a U-Net with depth-wise separable convolutional layers, attaining a dice coefficient of 0.54. A. G. Roy et al. [16] introduced ReLayNet, trained on weighted regression and dice loss, achieving a dice coefficient of 0.81.

In the OPTIMA challenge, two competitors used machine learning approaches to segment IRF from OCT images. L. De Sisternes et al. [17] extracted 34 quantitative features to segment IRF pixels, achieving an average dice coefficient of 0.7. Venhuizen et al. [18] proposed a multiscale CNN to segment IRF, performing retinal layer segmentation to remove detected fluids outside limiting layers, and achieved an average dice coefficient of 0.64.

The RETOUCH challenge aimed to segment IRF, SRF, and PED simultaneously. The leading team, Lu D et al. [19], initially segmented retinal layers using a graph-cut algorithm and trained a U-Net-based CNN to segment fluids, followed by random forest classification to detect falsely labelled pixels. The Rashno A. et al. [20] team employed a similar method, segmenting different layers using the graph-cut algorithm and training separate CNNs for each dataset vendor to segment PED, IRF, and SRF. MABIC32 proposed a two-stage cascaded U-Net, where the first U-Net segmented multiple fluids, and the second U-Net refined the segmentation masks for each fluid type.

Tennakoon et al. [21] developed a U-Net trained on patches of 3D OCT scans, using a novel loss function combining categorical cross-entropy, dice, and adversarial losses for simultaneous fluid segmentation. Apostolopoulos et al. [22] used retinal layer information to segment fluids efficiently, training a CNN combining dilated residual blocks in an asymmetric U-Net with individual binary cross-entropy losses for each fluid type. Helios et al. [23] proposed a two-stage CNN, where the first U-Net generated probability maps for fluid regions from generalized motion patterns, and the second U-Net refined the segmentation using combined inputs.

Morley et al. [24] implemented myopic warping with rotation-based augmentation to increase dataset volume and trained a U-Net based on ResNet to segment retinal fluids, using graph-cut and morphological operations for post-processing. Chen et al. [25] used a novel framework combining denoising, ROI location, Fast-RCNN for IRF segmentation, 3D region growing for SRF segmentation, and RPE layer segmentation for PED. Khaled et al. [26] employed various deep learning models like FCN, U-Net, and Deeplabv3+ for detecting retinal fluids using 2D and 2.5D OCT scans.

More recent advancements include novel approaches like the work by Lu et al. [27], who proposed a transformer-based model for OCT fluid segmentation, achieving state-of-the-art performance on several benchmark datasets. Additionally, a hybrid deep learning architecture combining convolutional neural networks (CNNs) and transformers by Zhang et al. [28] has shown promising results in segmentation accuracy and computational efficiency.

In recent advancements, cross-attention mechanisms have been shown to enhance segmentation performance by capturing the correlation between spatial and temporal features, as demonstrated in the Temporal–Spatial Cross-Attention Network (TSCA-Net) for BCI signal decoding [29]. Similarly, the Dual Cross-Attention (DCA) [30] module addresses the semantic gap between encoder and decoder in U-Net architectures for medical image segmentation by using cross-attention across both channel and spatial dimensions. Integrating such attention mechanisms improves multiscale feature fusion, enhancing segmentation accuracy with minimal computational overhead, and making them valuable for precision tasks like subretinal fluid segmentation in OCT. Also, advancements in Graph Neural Networks (GNNs), such as the Layer-wise Self-adaptive GAT (LSGAT), have addressed the over-smoothing issue in deeper Graph Attention Networks (GATs) by adaptively adjusting attention coefficients, enhancing performance in node classification tasks [31]. Similarly, the Graph Attention U-Net (GA-U-Net) [32] has demonstrated effectiveness in medical imaging, particularly in retinal layer surface detection and choroidal neovascularization (CNV) segmentation in OCT images. The GA-U-Net integrates topological and pathological knowledge through graph attention mechanisms, improving feature embedding and segmentation accuracy in the context of retinal diseases. These models highlight the growing potential of graph attention in both node-based tasks and complex medical image segmentation.

In the RETOUCH challenge, leading teams utilized retinal-layer information and trained networks on either patch-wise data or C scans to boost performance. Many teams trained separate networks for each fluid type and employed cascaded networks to refine probability maps, which increased the complexity of training and required loading multiple networks during inference.

To address these issues, we proposed a novel deep learning architecture, trained on 2D OCT slices to enhance performance in segmenting multiple fluid classes. The proposed network generates novel edge maps of OCT slices and uses them as dual inputs. Unlike other edge attention networks, our model incorporates spatial and edge attention modules at every level, overcoming blurry and smooth edges by enhancing network representation ability. Integrated residual and inception modules improve performance, particularly for fluids with varying shapes and sizes. Additionally, separable convolution layers with autoencoder-based attention refine feature extraction and localization.

## 3. Materials and Methods

This Section discusses the material and methodology of the proposed work, such as pre-processing techniques, implementation of Hybrid-U-NET, loss functions, and performance matrices, in detail.

### 3.1. Dataset

This study utilizes three publicly available datasets: RETOUCH [33], OPTIMA [34], and DUKE DME [35]. These datasets provide various OCT scans from multiple vendors, ensuring robust training and evaluation of the proposed segmentation network.

RETOUCH Dataset: The RETOUCH dataset, released as part of the MICCAI 2017 challenge, focuses on comparing algorithms for segmenting IRF, SRF, and PED from OCT scans across different vendors. The training set includes 70 C scans: 24 Cirrus, 24 Spectralis, and 22 Topcon, with pixel-level annotations for each fluid type. The testing set contains 42 C scans without pixel-level annotations. As a result, the RETOUCH training dataset is used for both training and testing the proposed model. This dataset includes imaging from both Diabetic Macular Oedema (DME) and Age-related Macular Degeneration (AMD).

OPTIMA Dataset: The OPTIMA dataset is part of the OPTIMA Cyst Segmentation Challenge from MICCAI 2015. This challenge aims to segment IRF from OCT scans provided by vendors such as Cirrus, Spectralis, Nidek, and Topcon. This dataset similarly contains imaging for both DME and AMD, covering segmentation tasks for both intraretinal and subretinal fluids across different disease stages. The dataset includes 15 scans for training, eight scans for Stage 1 testing, and seven scans for Stage 2 testing. Each scan is accompanied by pixel-level annotation masks created by two different graders.

DUKE DME Dataset: The DUKE dataset focuses on DME, specifically on intraretinal fluid segmentation, which is crucial for this particular disease. The DUKE DME dataset consists of 10 Spectralis C scans, with pixel-level annotations for IRF provided by two graders. These annotations are available for 11 B scans from each C scan, resulting in 110 annotated B scans. These annotated scans are utilized for both training and testing purposes.

### 3.2. Pre-Processing

**Figure 2:**
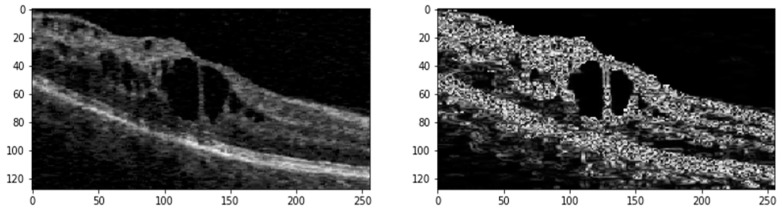
ROI generated from the Original OCT scan (**Left**) and its Edge map (**Right**).

OCT scans from different datasets are generally 3D (C scans), offering volumetric views of retinal layers. In contrast, B scans are 2D cross-sectional slices taken along a horizontal or vertical axis of the retina, with one axis showing the slice and the other depicting depth. For this study, we extracted B scans from the C scans, along with their corresponding pixel-annotated ground truth masks, to form a 2D image dataset. Regardless of the vendor—whether Cirrus, Spectralis, Nidek, or Topcon—B scans typically contain varying amounts of speckle noise and exhibit contrasting differences. We randomly select one with better contrast from all the extracted B scans. We then apply histogram matching across the entire dataset using this selected B scan as a reference. This process helps standardize the intensity distribution and enhances the contrast of the B scans. The images are subjected to median filtering to reduce random noise following histogram matching. The B scans typically contain a small area of interest (ROI) within the image. To eliminate unnecessary background, we calculate the entropy of the images, filtering out regions that do not contain retinal layers or fluids. This pre-processing step ensures that the data fed into the deep learning model are of high quality and consistency, enhancing the overall segmentation performance. Figure 2 shows the ROI generated from the original image with its edge map after pre-processing.

The pre-processed images (Ι*m*) undergo further refinement using a median filter. Following this, contrast-limited Adaptive Histogram Equalization (CLAHE) with a clip limit (ℒ) of 1.37 and gamma correction (g) with a gamma value (*γ*) of 1.7 is applied to enhance image contrast. Additionally, a linear filter (*ψ*) based on a multivariate Taylor series with antisymmetric properties is used with a *τ* value of 1.8 to generate edge maps of the denoised images.

### 3.3. Proposed Hybrid Deep Learning Architecture

**Figure 3:**
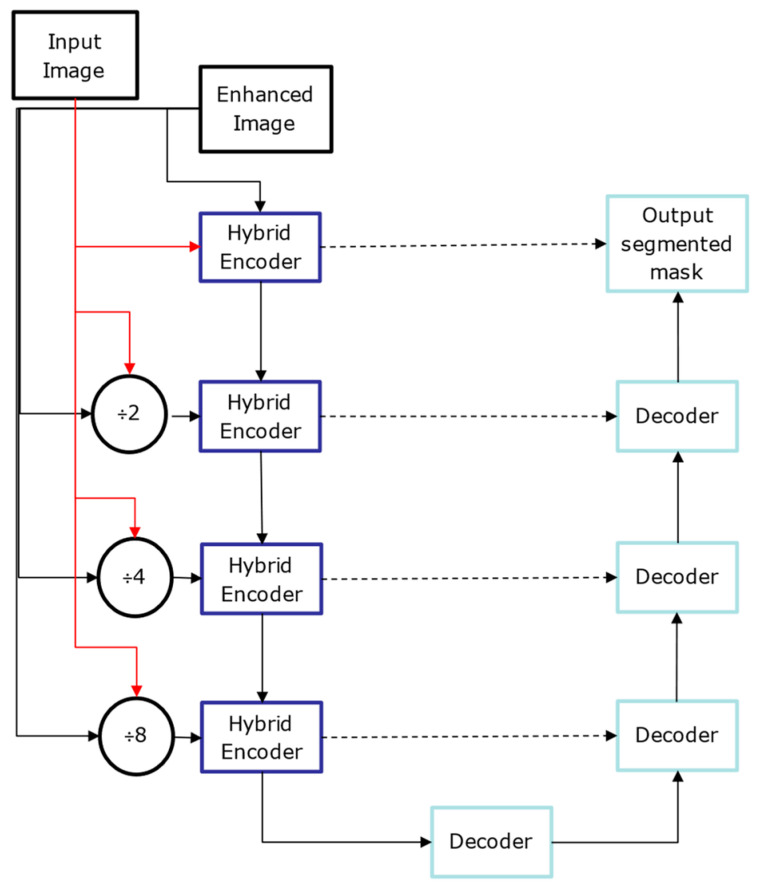
Proposed multiscale attention-based U-Net Model.

**Figure 4:**
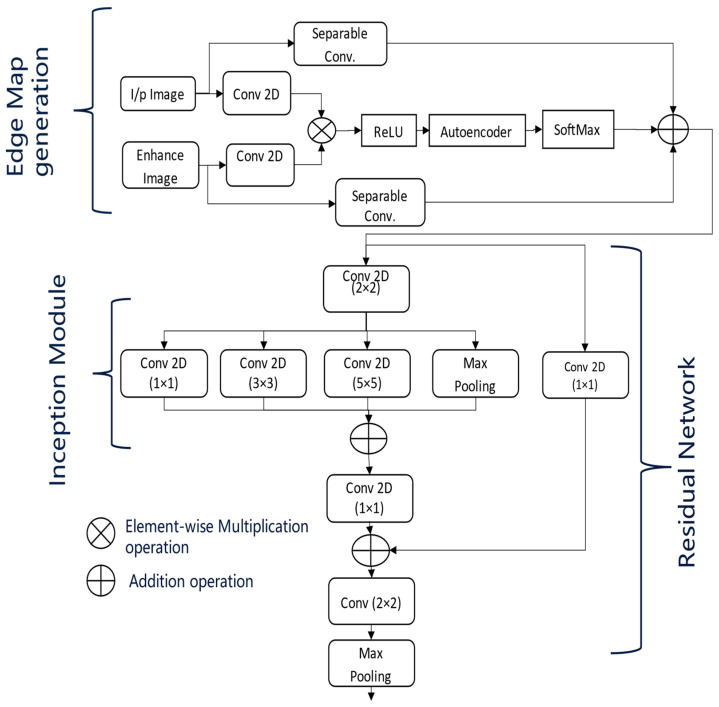
Detailed Architecture of Encoder block of Proposed Architecture.

The proposed network is based on an encoder–decoder architecture inspired by U-Net [7], as illustrated in Figure 3. In Figure 3, the dotted arrow represents a skip connection and solid lines with different colours are used to differentiate two lines from each other. This architecture processes the enhanced input images along with their corresponding edge maps. At each stage of the encoder, the dimensions of the input images are reduced by half, with the down-sampled images being passed on to the subsequent layers. The encoders utilize the inception and residual sections, along with an Unet-based multiscale attention mechanism, as described in Figure 4.

The multiscale attention mechanism first applies separate convolutions to the pre-processed input images and their edge maps. The resulting feature maps are then multiplied elementwise, followed by activation with the ReLU function.

These feature maps are subsequently processed by an autoencoder, allowing the network to learn compact feature representations that act as a self-attention mechanism. Afterwards, a softmax activation is applied, and the feature maps generated from the separable convolutions of the input images and edge maps are then concatenated.

At the end of each encoder stage, convolution operations are performed on the feature maps generated by the attention module, as shown in Figure 4. This step is crucial for extracting multiscale features necessary for effective retinal fluid segmentation. The Inception module captures features at various scales using convolutional kernels of different sizes (one, three, and five), while the residual module maintains the flow of information across multiple convolutional layers. Finally, max pooling reduces the feature map dimensions by half, preparing them for the next stage of the encoder.

At the final encoder stage, the feature maps are up-sampled to double their dimensions, preparing them for input into the decoder. Before concatenating the feature maps from the decoder with the corresponding encoder via skip connections, a convolution operation is performed. Subsequent convolution and up-sampling operations refine the concatenated feature maps.

In the last decoder stage, a final convolutional layer is added, followed by a sigmoid activation function. The number of kernels in this layer matches the number of different fluid classes, producing the final segmentation masks.

The depicted network architecture in Figure 3 is designed for OCT image segmentation and incorporates an advanced encoder block. The model takes two input images: the original OCT image and its enhanced version, which has been pre-processed to improve contrast and reduce noise. Initially, separate 2D convolutions are applied to both the original and enhanced images. The resulting feature maps are then combined using elementwise multiplication and passed through a ReLU activation function to introduce non-linearity.

This combined feature map is further processed by an autoencoder, which performs encoding and decoding steps to learn condensed feature representations. The autoencoder output is then subjected to a SoftMax activation, producing a probability distribution over the possible segmentation classes. A separable convolution is applied to refine these feature maps. The architecture follows an encoder path integrating Residual [36] and Inception modules [37]. The Inception modules perform convolutions with different kernel sizes (1 × 1, 3 × 3, and 5 × 5) to capture features at multiple scales. Max pooling layers are included to reduce the spatial dimensions while retaining significant features. The outputs from these convolutions and the max pooling layer are combined using elementwise addition, followed by another 1 × 1 convolution to integrate the combined feature maps. Further refinement is achieved with a 2 × 2 convolution, and additional max pooling reduces the dimensions further. The architecture includes skip connections, linking earlier layers to later layers to ensure better gradient flow during backpropagation and an effective combination of low-level and high-level features. Inspired by U-Net, this network architecture leverages dual input processing, an autoencoder-based attention mechanism, multiscale feature extraction through Inception modules, and residual connections. These components work together to segment retinal fluids in OCT images effectively, enhancing the model’s ability to capture detailed and varied features crucial for accurate segmentation.

## 4. Experimental Setup

The proposed hybrid deep learning architecture for retinal fluid segmentation was trained using a set of carefully selected hyperparameters. The initial learning rate was set to 0.001, adjusted dynamically using a scheduler that reduced the rate by a factor of 0.1 upon detecting a plateau in validation performance. A batch size of 16 was chosen to balance computational efficiency and training stability. The Adam optimizer, known for its effectiveness in handling sparse gradients and adaptive learning rates, was utilized. The loss function combined binary cross-entropy with Dice loss to ensure pixel-wise accuracy and segmentation quality. The network was trained for up to 100 epochs, with early stopping based on validation loss to prevent overfitting. L2 regularization with a coefficient of 0.0001 was applied to the convolutional layers to mitigate overfitting further. Data augmentation techniques were employed to enhance model robustness, including random rotations, flips, and intensity variations.

During training, the dataset from each source (https://www.kaggle.com/datasets/saivikassingamsetty/retouch, https://www.kaggle.com/datasets/paultimothymooney/kermany2018/data, https://www.kaggle.com/datasets/paultimothymooney/chiu-2015) (Accessed on 10 October 2024)) was split into training and validation sets in an 80-20 ratio. Input images were normalized to have zero mean and unit variance. The training loop processed data in mini-batches, calculating the loss and backpropagating gradients to update network weights. Validation was conducted after each epoch, allowing for learning rate adjustments and monitoring of performance metrics. This rigorous training procedure ensured the network achieved high accuracy and reliability in segmenting retinal fluids from OCT images.

## 5. Results and Discussion

In this Section, we’ll discuss our segmentation results on various datasets. Additionally, we will compare our hybrid U-Net with existing methods to comprehensively understand its performance. In all the test images, red indicates IRF, blue indicates PED, and green indicates SRF5.1. Performance Metrices

### 5.1. Performance Metrices

The result of the proposed architecture is evaluated using performance metrics like Precision, Recall, Area Under the Curve (AUC), and F1 Score. All these metrics were calculated using the following formulae:

(1) $$Precision=\frac{TP}{TP+FP}$$

(2) $$Recall=\frac{TP}{TP+FN}$$

(3) $$F1\;Score=\frac{2\times precision\times recall}{precision+recall}$$

TP represents True Positives, FP represents False Positives, and FN represents False Negatives. We have also evaluated our model using the Dice Coefficient, which measures the area of overlap between two masks.

Among these metrics, Precision is particularly important in clinical settings, as it helps to minimize false positives (incorrectly identified subretinal fluids). High precision ensures that the regions identified as fluid are indeed true fluid regions, which is crucial for physicians when diagnosing conditions like Diabetic Macular Oedema (DME) or Age-related Macular Degeneration (AMD).

While Precision is critical, the F1 Score provides a balance between Precision and Recall, which is valuable for ensuring that both false positives and false negatives are minimized. Therefore, the F1 Score offers a more holistic view of model performance, particularly when both error types are of concern. Additionally, AUC allows us to assess the overall performance across different decision thresholds, making it useful in model evaluation.

### 5.2. Results on RETOUCH Dataset

The provided Table 1 details the performance metrics—Precision (Pr), Recall (Re), and F1 Score (F1)—for segmenting intraretinal fluid (IRF), subretinal fluid (SRF), and pigment epithelial detachment (PED) using OCT scans from three vendors: Cirrus, Spectralis, and Topcon. The metrics for each fluid type and the overall averages across all vendors are presented.

For Cirrus scans, the segmentation network demonstrates balanced performance with an F1 Score of 0.82 for IRF and exceptional performance for SRF and PED, achieving F1 Scores of 0.93 and 0.94, respectively. This results in high average metrics, with Precision, Recall, and F1 Score of 0.90, indicating robust and reliable segmentation. Spectralis scans also show balanced performance for IRF with an F1 Score of 0.81 and excellent performance for SRF, achieving the highest F1 Score of 0.95 among all metrics. The PED segmentation is consistent with an F1 Score of 0.91, leading to overall averages of 0.90 for Precision, 0.88 for Recall, and 0.89 for F1 Score, suggesting reliable segmentation with slight variation in recall. Topcon scans exhibit slightly lower performance compared to Cirrus and Spectralis. The IRF segmentation has a lower Precision of 0.69, resulting in an F1 Score of 0.74. SRF and PED segmentation show moderate performance with F1 Scores of 0.89 and 0.92, respectively.

The overall averages for Topcon are 0.84 for Precision, 0.86 for Recall, and 0.85 for F1 Score, indicating slightly lower but still effective performance. The segmentation network achieves a well-balanced and robust performance across different vendors and fluid types, with an overall average Precision, Recall, and F1 Score of 0.88.

**Figure 5:**
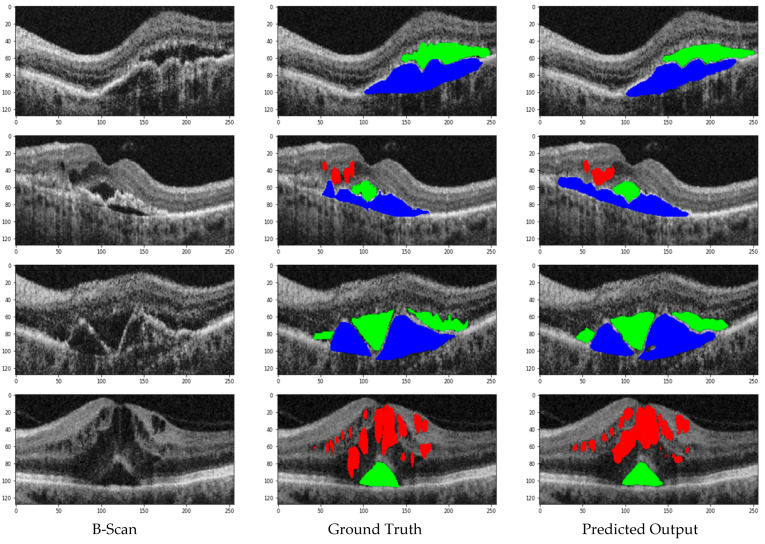
Retinal fluid Segmentation using the proposed model on the RETOUCH.

**Figure 6:**
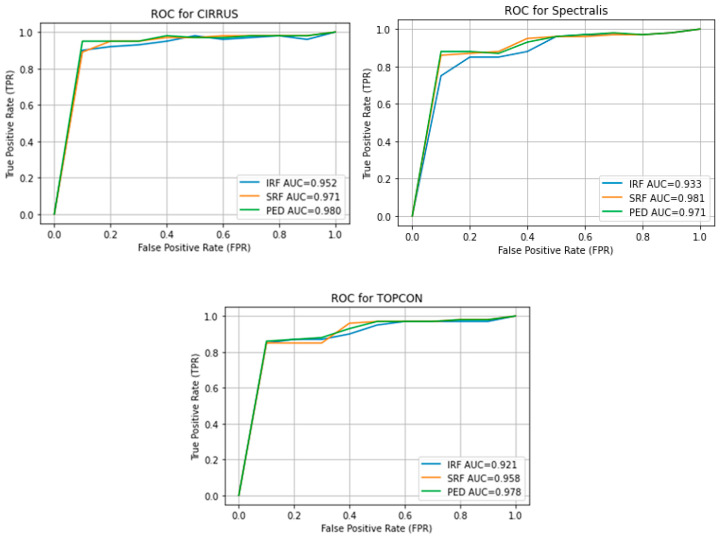
ROC curve for RETOUCH dataset from various vendors.

The high average F1 Scores and balanced Precision and Recall metrics confirm the network’s robustness in accurately identifying and segmenting IRF, SRF, and PED in OCT images. Cirrus and Spectralis devices show the highest performance, while Topcon demonstrates slightly lower performance yet still effective segmentation capabilities. Retinal fluid Segmentation using the proposed model on the RETOUCH dataset is given in Figure 5. Figure 6 shows the Receiver Operating Characteristics (ROC) of the retouch dataset with various vendors with corresponding Areas under Curve (AUC).

### 5.3. Results on OPTIMA Dataset

The performance metrics for the OPTIMA dataset, as detailed in Table 2, indicate the effectiveness of the segmentation model across different vendors and ground truth annotationsFor Cirrus, the model shows moderate performance for Ground Truth 1 (Precision: 0.78, Recall: 0.71, F1 Score: 0.74) and improved metrics for Ground Truth 2 (Precision: 0.84, Recall: 0.81, F1 Score: 0.82). Nidek exhibits strong performance for Ground Truth 1 (Precision: 0.83, Recall: 0.77, F1 Score: 0.80) but shows a drop in Recall for Ground Truth 2, leading to a lower F1 Score of 0.73. Spectralis maintains a balanced performance with F1 Scores of 0.76 and 0.71 for the two ground truths, respectively. Topcon shows high Precision for both ground truths (0.86 and 0.91), but lower Recall (0.69 and 0.71), resulting in F1 Scores of 0.77 and 0.80. As the F1 Score is a balance between Precision and Recall, these results suggest that while the model is precise and accurate in identifying true positives, it may miss some true positives, leading to lower recall values

**Figure 7:**
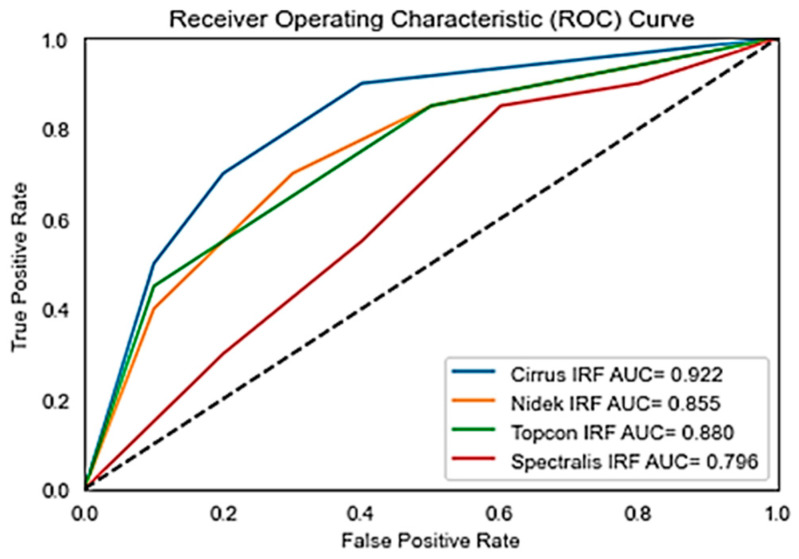
ROC Curve for OPTIMA Dataset with AUC.

**Figure 8:**
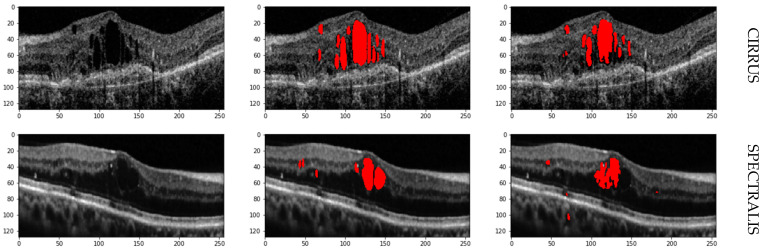

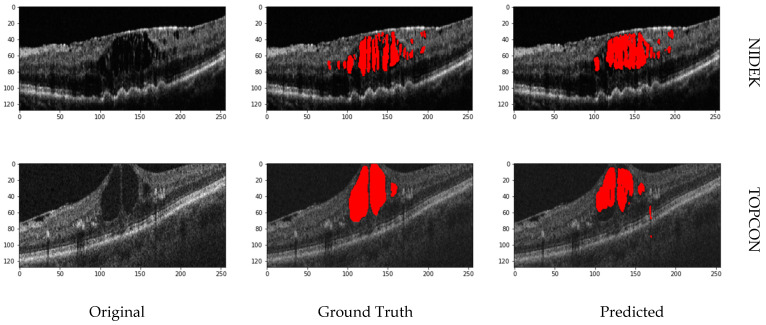
Prediction on OPTIMA Dataset.

Overall, the analysis highlights that the model performs variably across vendors, with Cirrus and Topcon showing more consistent high performance, while Nidek and Spectralis exhibit more variability, especially in Recall. Further optimization is needed for consistent performance across all vendors. Further comparison of our proposed model in terms of F1 score, with state of the art is given in Table 3. Figure 7 shows the receiver operating curve for the OPTIMA dataset with all vendors and its corresponding Area under Curve (AUC). While Precision, Recall, and F1 Scores give us a snapshot of a model’s performance at a specific threshold, AUC provides a broader view. Figure 8 shows the prediction on the OPTIMA dataset from various vendors using the proposed model.

### 5.4. Results on Duke Dataset

**Figure 9:**
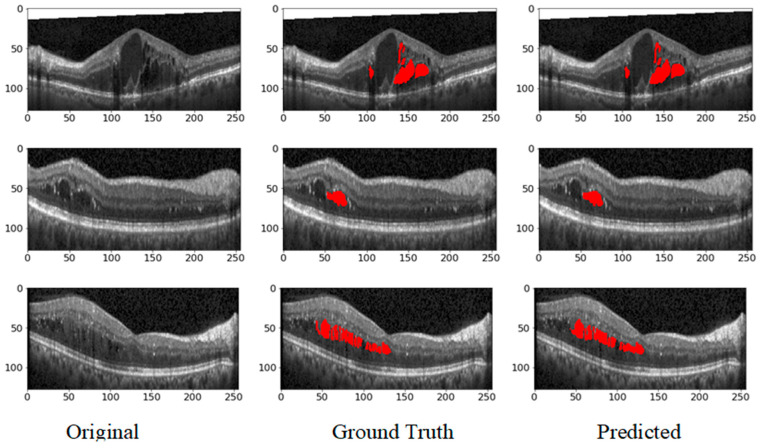
Predicted output from DUKE dataset.

**Figure 10:**
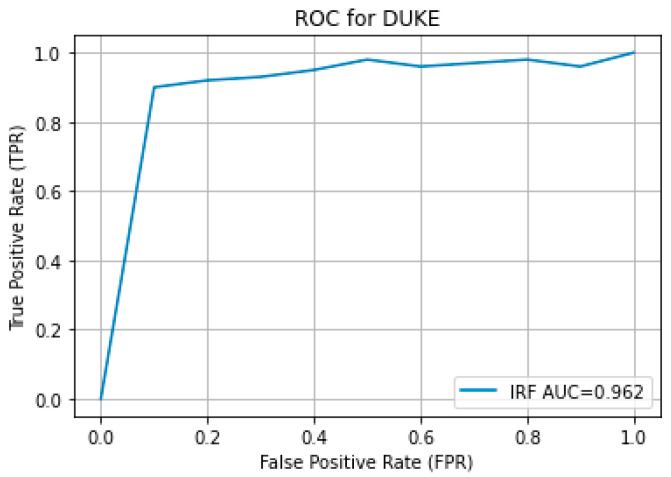
ROC curve for DUKE DSC.

The results obtained from the proposed hybrid deep learning architecture for the DUKE dataset are promising, demonstrating high performance in segmenting retinal fluids. The model achieved a Precision of 0.91, indicating a high level of accuracy in correctly identifying positive instances. The Recall of 0.88 suggests that the model effectively captures most of the true positives. The F1 Score, which balances precision and recall, stands at 0.89, reflecting robust overall performance. These metrics indicate that the proposed architecture is highly effective in segmenting retinal fluids in OCT images from the DUKE dataset. Figure 9 showcases the predicted output, illustrating the model’s capability to generate accurate and reliable segmentation masks. The high precision and recall values highlight the model’s ability to identify retinal fluids accurately and ensure that most of the relevant instances are detected, making it a valuable tool for clinical applications in retinal disease diagnosis and treatment planning. The ROC curve for the DUKE DSC database with the corresponding AUC is given in Figure 10.

### 5.5. Discussion

The proposed multiscale attention-based U-Net architecture shows strong segmentation results for intraretinal fluid (IRF), subretinal fluid (SRF), and pigment epithelial detachment (PED) across the RETOUCH, OPTIMA, and DUKE datasets. However, performance varies by imaging vendor, reflecting the challenges of achieving consistent results across different systems. In the RETOUCH dataset, which includes AMD and DME cases, Cirrus and Spectralis devices demonstrated the highest performance, especially for SRF and PED, with F1 Scores above 0.90. However, Topcon showed lower performance, particularly for IRF segmentation (F1 Score: 0.74), suggesting potential limitations in handling this fluid type with Topcon scans. Despite these differences, the overall results indicate the model’s robustness in identifying and segmenting retinal fluids for clinical applications. For the OPTIMA dataset, Cirrus and Topcon again showed consistent performance, with F1 Scores up to 0.82, while Nidek and Spectralis displayed more variability, especially in Recall. This variation indicates that while the model is effective, further tuning is needed to ensure consistent detection of true positives, particularly for more challenging cases in DME. In the DUKE dataset, focused on DME, **Spectralis** scans achieved strong results with an F1 Score of 0.89, confirming the model’s effectiveness for DME segmentation. The high precision and recall values highlight the architecture’s potential for accurate clinical diagnosis.

Overall, the architecture demonstrates high accuracy across datasets, particularly with Cirrus and Spectralis, but performance variability across vendors like Topcon and Nidek suggests the need for further optimization. Achieving consistent performance across all platforms remains a challenge, but the model’s strong results indicate its potential for aiding in the clinical management of retinal diseases like AMD and DME.

## 6. Conclusions

In this research article, we addressed the critical challenges associated with the segmentation of retinal fluids in patients with retinal diseases such as Diabetic Macular Oedema (DME) and Age-related Macular Degeneration (AMD). These conditions often lead to fluid accumulation in various retinal layers, making precise segmentation essential for accurate diagnosis and effective treatment planning. Despite significant advancements in automated segmentation techniques, existing methods struggle with accurately segmenting multiple fluid types and maintaining edge precision.

To overcome these limitations, we proposed a robust deep-learning architecture that uses B-scan images from various datasets to segment multiple classes of retinal fluids. Our novel approach involves generating edge maps of OCT slices and using dual inputs to enhance segmentation accuracy. This method is integrated into an autoencoder-based hybrid network, which combines the strengths of Residual and Inception modules within the encoder section. These modules are specifically designed to extract multiscale and multilevel features, significantly improving the performance of semantic segmentation of retinal fluids.

Our proposed method has shown promising results, with significant improvements in segmentation accuracy and edge precision compared to existing techniques. This advancement is crucial for clinical applications, providing a more reliable tool for diagnosing and managing retinal diseases. By improving the accuracy and efficiency of retinal fluid segmentation, our hybrid deep learning architecture can enhance patient clinical outcomes, contributing to better treatment strategies and overall eye health management.

In conclusion, developing and validating our deep learning-based segmentation model represents a significant step forward in retinal imaging. Our approach addresses the challenges in retinal fluid segmentation, offering a robust and accurate solution that can be integrated into clinical practice to improve patient care.

## Figures and Tables

**Table 1:** Performance Metrics for Retouch Dataset.

Vendor	IRF			SRF			PED			Average		
	Pr	Re	F1	Pr	Re	F1	Pr	Re	F1	Pr	Re	F1
**Cirrus**	0.81	0.83	0.82	0.94	0.93	0.93	0.95	0.93	0.94	0.9	0.90	0.90
**Spectralis**	0.83	0.79	0.81	0.97	0.94	0.95	0.91	0.92	0.91	0.90	0.88	0.89
**Topcon**	0.69	0.79	0.74	0.91	0.87	0.89	0.92	0.93	0.92	0.84	0.86	0.85
**Average**	0.78	0.80	0.79	0.94	0.91	0.93	0.93	0.93	0.93	0.88	0.88	0.88

**Table 2:** Performance Metric for OPTIMA Dataset.

Vendor	Ground Truth	Precision	Recall	F1score
**Cirrus**	1	0.78	0.71	0.74
	2	0.84	0.81	0.82
**Nidek**	1	0.83	0.77	0.80
	2	0.82	0.66	0.73
**Spectralis**	1	0.79	0.73	0.76
	2	0.74	0.69	0.71
**Topcon**	1	0.86	0.69	0.77
	2	0.91	0.71	0.80

**Table 3:** Comparison of Proposed Method (F1 Score) with the existing method.

Vendor	GT	[17]	[18]	[5]	[38]	[39]	Proposed Method
Cirrus	1	0.65	0.61	0.57	0.14	0.62	0.74
Cirrus	2	0.61	0.56	0.66	0.14	0.62	0.82
Nidek	1	0.73	0.55	0.59	0.41	0.77	0.80
Nidek	2	0.70	0.52	0.57	0.41	0.76	0.73
Spectralis	1	0.66	0.60	0.56	0.25	0.71	0.76
Spectralis	2	0.67	0.61	0.55	0.25	0.72	0.71
Topcon	1	0.73	0.72	0.59	0.21	0.75	0.77
Topcon	2	0.67	0.61	0.55	0.25	0.77	0.80

## Data Availability

Data source is cited in within the article.
